# Graph and Multi-Level Sequence Fusion Learning for Predicting the Molecular Activity of BACE-1 Inhibitors

**DOI:** 10.3390/ijms26041681

**Published:** 2025-02-16

**Authors:** Shaohua Zheng, Changwang Zhang, Youjia Chen, Meimei Chen

**Affiliations:** 1 College of Physics and Information Engineering, Fuzhou University, Fuzhou 350108, China; 2 College of Traditional Chinese Medicine, Fujian University of Traditional Chinese Medicine, Fuzhou 350122, China

**Keywords:** Alzheimer’s disease, BACE-1 inhibitor, molecular activity prediction, graph neural network, fusion learning

## Abstract

The development of BACE-1 (β-site amyloid precursor protein cleaving enzyme 1) inhibitors is a crucial focus in exploring early treatments for Alzheimer’s disease (AD). Recently, graph neural networks (GNNs) have demonstrated significant advantages in predicting molecular activity. However, their reliance on graph structures alone often neglects explicit sequence-level semantic information. To address this limitation, we proposed a Graph and multi-level Sequence Fusion Learning (GSFL) model for predicting the molecular activity of BACE-1 inhibitors. Firstly, molecular graph structures generated from SMILES strings were encoded using GNNs with an atomic-level characteristic attention mechanism. Next, substrings at functional group, ion level, and atomic level substrings were extracted from SMILES strings and encoded using a BiLSTM–Transformer framework equipped with a hierarchical attention mechanism. Finally, these features were fused to predict the activity of BACE-1 inhibitors. A dataset of 1548 compounds with BACE-1 activity measurements was curated from the ChEMBL database. In the classification experiment, the model achieved an accuracy of 0.941 on the training set and 0.877 on the test set. For the test set, it delivered a sensitivity of 0.852, a specificity of 0.894, a MCC of 0.744, an F1-score of 0.872, a PRC of 0.869, and an AUC of 0.915. Compared to traditional computer-aided drug design methods and other machine learning algorithms, the proposed model can effectively improve the accuracy of the molecular activity prediction of BACE-1 inhibitors and has a potential application value.

## 1. Introduction

Alzheimer’s Disease (AD) is a chronic and irreversible neurodegenerative disease characterized by the deposition of amyloid plaques in the brain, which are significant because they disrupt cell function and are considered one of the key pathological markers of AD [1,2]. According to the amyloid hypothesis, the pathogenesis of AD is closely related to the increased aggregation of amyloid β (Aβ), which is produced by the cleavage of the amyloid precursor protein by β-Secretase 1 (BACE-1) [3]. Therefore, methods to inhibit the BACE-1 catalysis of Aβ aggregation have become an effective strategy for treating AD patients. Studies have shown that BACE-1 plays a critical role in the formation of Aβ in AD, and that the concentration and activity of BACE-1 were significantly increased in the brains and bodily fluids of AD patients [4,5]. Therefore, BACE-1 is considered a primary drug target for reducing early Aβ production in AD, and methods to inhibit the BACE-1 catalysis of Aβ aggregation have become an effective strategy for treating AD patients, demonstrating significant therapeutic potential.

With the advancement of computer technology, computer-aided drug design has been extensively utilized for predicting the activity of BACE-1 inhibitors, significantly improving the efficiency of drug screening. Ponzoni et al. [6] and Bao et al. [7] used the QSAR classification model to predict AD-associated β-secretase inhibitor (BACE1) activity. Huang et al. [8] and Aswathy et al. [9] adopted 2D-QSAR, HQSAR, and 3D QSAR modeling approaches to identify the structural and physicochemical requirements for the potential Aβ aggregation inhibition. It can be seen that the development of BACE-1 inhibitors for AD heavily relies on virtual screening techniques such as QSAR models and molecular docking. However, designing an effective QSAR model is a challenging task. In addition, traditional machine learning algorithms such as SVM, RF, KNN, NB, and XGBoost are widely used in the classification of BACE-1 inhibitors [10]. However, these methods often rely on manually crafted molecular descriptors or molecular fingerprint features. Molecular descriptors quantitatively represent a molecule’s properties, such as molecular weight, atom count, and complex attributes. Crafting these descriptors requires expert knowledge and may not fully capture all of the molecular interactions. Molecular fingerprints, on the other hand, are binary vectors that indicate the presence or absence of specific substructures. Although computationally efficient, fingerprints might miss subtle but crucial differences essential for certain biological interactions.

In recent years, graph neural networks (GNNs) have been extensively studied and applied in the fields of molecular property prediction and drug discovery. GNNs represent molecules as graph structures, where atoms are depicted as nodes and chemical bonds as edges, effectively capturing the topology and local environment of molecules. During training, GNNs learn complex relationships between nodes and their surrounding neighborhoods by aggregating and updating node information. This approach has shown significant advantages and potential in enhancing molecular property prediction and advancing drug discovery efforts. Rong et al. [11] presented a new framework named GROVER (Graph Representation frOm self-superVised mEssage passing tRansformer) for self-supervised graph converter learning on large-scale molecular data. Deng et al. [12] developed an integrated framework XGraphBoost to extract the features using a GNN and build an accurate prediction model of molecular properties using the classifier XGBoost. Zhang et al. [13] proposed a fragment-oriented multi-scale graph attention model for molecular property prediction. Choo et al. [14] devised a fingerprint-enhanced graph attention network (GAN) for antibiotic discovery. Han et al. [15] formulated a hierarchical molecular representation using both atomic and motif-level features, leveraging a GNN and a transformer-based module for enhancing property prediction. Bongini et al. [16] introduced a composite GNN by using a multi-state updating mechanism to process heterogeneous molecular graphs, which was more efficient at extracting information and improving molecular property prediction tasks compared to traditional GNNs.

On the other hand, significant progress has also been made in molecular representation learning using SMILES (Simplified Molecular Input Line-Entry System) sequence information. Xu et al. [17] offered a seq2seq fingerprint model with an unsupervised deep molecular embedding for drug discovery. Honda et al. [18] presented a SMILES–Transformer model with pre-trained molecular fingerprint for low data drug discovery. Wang et al. [19] proposed a SIMLES–BERT model with large scale unsupervised pre-training for molecular property prediction. Kim et al. [20] designed a self-supervised method in which both SMILES and the chemical context were learned during Transformer pre-training. The key lies in the adjacency matrix embedding and learning structure, which can infer chemical descriptors through drug similarity estimation. Tran et al. [21] developed a semi-supervised learning model that combines SMILES strings, utilizing pre-training and fine-tuning strategies, and incorporating 3D compound structures for attention scores to improve the prediction of anti-malaria drug candidates. Zheng et al. [22] suggested a BERT-based model for pre-training on SMILES sequences, tackling the issues of low contextual dependence of SMILES symbols and the existence of multiple SMILES representations for the same compound. However, these methods typically emphasized molecular graph structures or the semantic information derived from SMILES sequences, but they fell short of fully leveraging the global information within molecules and their intrinsic complex relationships. As a result, there remains a need to enhance the representation of the molecules’ overall characteristics.

To address these challenges, we developed a dual-modality fusion framework integrating molecular graph representations with a SMILES sequence analysis for an enhanced prediction of compound bioactivity, specifically targeting BACE-1 inhibition. For encoding molecular graph structures, we employed a multi-GNN enhanced with atomic-level feature attention mechanisms to more effectively capture the topology and local information of molecules. For encoding the SMILES character information, we designed a BiLSTM–Transformer framework with a hierarchical attention mechanism to encode multi-level substrings, enhancing the understanding of molecular structural details. The main contributions of our method were summarized as follows:

We developed a graph encoding model with an atomic-level feature attention mechanism. The model not only highlighted the key atoms and chemical bonds in the molecular graph structure, but also further strengthened the feature representation ability of the molecular graph.

We designed a BiLSTM–Transformer framework enhanced with a hierarchical attention mechanism to encode substrings derived from SMILES strings. These substrings were categorized at the functional group, ion level, and atomic level, which can enhance the understanding of molecular structure details and consequently improve the prediction accuracy.

Common functional group substructures were extracted from the dataset to generate unique sequence markers, so that the encoded molecular substructures can enrich more detailed information, so as to reflect the functional properties of molecules more accurately. Moreover, the generated labeling sequence was shorter than the traditional atomic labeling, which can reduce the computational cost.

The structure of this paper is as follows: Section 2 introduces the methods used in detail, Section 3 presents the experimental results and discussion, and Section 4 summarizes the main conclusions of this study.

## 2. Results and Discussion

We employed the PyTorch deep learning framework and the PyTorch Geometric library to develop the GSFL model. The training process was executed end-to-end with the Adam optimizer (Adaptive Moment Estimation) [23] for parameter updates. Adam is a widely used optimization algorithm in deep learning because it combines the advantages of RMSProp and Momentum, offering an adaptive learning rate that automatically adjusts during training. In addition, we conducted experiments using ten different random seeds to validate the effectiveness of the proposed method. These experiments included hyperparameter tuning, comparison studies, and ablation tests. Random seeds are crucial in deep learning experiments, as they ensure the reproducibility and robustness of results. Choosing ten random seeds was a balanced decision, taking into account statistical reliability, computational resources, time constraints, and industry standards.

### 2.1. Hyperparameter Tuning

In the algorithm implementation process, this study determined the following ranges for the hyperparameters: a batch size of 32 or 64; a learning rate of 1 × 10^−3^, 1 × 10^−4^, or 1 × 10^−5^; the number of GNN layers and sequence network layers as 3 or 5; the hidden layer dimensions of the graph encoder (GNN encoder hidden layer dimension) and sequence encoder (sequence encoder hidden layer dimension) as 32, 64, and 128, and 64, 128, and 256, respectively; and a dropout rate of 0.3, 0.5, or 0.7. These hyperparameters were carefully selected and validated through extensive experiments, aiming to optimize the model’s performance and enhance its generalization capability.

Then, we assessed model performance under different hyperparameter settings using a 10-fold cross-validation, ensuring that every data point had a chance to be a part of the test set, which provided a comprehensive evaluation of model performance. Additionally, we employed a stratified cross-validation to address class imbalance, ensuring that the proportion of each class in every fold remained consistent. Through this comprehensive approach, we identified the hyperparameter configuration that achieved a good balance across multiple key metrics, realizing an optimal trade-off between the overall model performance and the ability to identify the minority class. In the hyperparameter tuning process, we aimed to optimize Acc while also considering the F1-score and MCC, particularly given the class imbalance in the dataset. This thorough method allowed us to determine the hyperparameter configuration that best balanced the overall model performance and minority class identification. Ultimately, we selected the combination that provided the optimal trade-off among these metrics, resulting in the previously mentioned hyperparameter settings.

The best-performing hyperparameter configuration was as follows: a batch size of 32, a learning rate of 1 × 10^−4^, 3 layers for both the GNNs and the sequence network, a hidden layer dimension of 64 for the GNNs, a hidden layer dimension of 128 for the sequence network, and a dropout rate of 0.5. These hyperparameters were carefully tuned to maximize the overall model performance.

### 2.2. Comparison Results and Discussion

In this work, we compared our experimental results with those of three recently proposed models for predicting BACE-1 inhibitor activity: RF (Random Forest) [7], XGraphBoost [11], and FraGAT [13]. Additionally, we calculated experimental results using several classical machine learning algorithms: SVM, KNN, XGBoost, and CNN, with code sourced from reference [6]. In experimental comparisons, we ensured that all the above-mentioned baseline model parameters were locally optimized to ensure fairness. The main parameters and results involved in the optimization are described below as follows:

SVM (Support Vector Machine): The optimized parameters include the kernel function (kernel), regularization coefficient (C), and kernel function parameter (gamma). The optimization method is Grid Search combined with a 5-fold cross-validation, and the optimal parameters for the final: {‘kernel’: ‘rbf’, ‘C’: 10, ‘gamma’: 0.01}.

KNN (K-Nearest Neighbors): The optimized parameters include the number of neighbors (n_neighbors), distance metric (‘metric’), and weight strategy (‘weights’). The optimization method is Grid Search combined with a 5-fold cross-validation, and the optimal parameters for the final: {‘n_neighbors’: 5, ‘metric’: ‘manhattan’, ‘weights’: ‘distance’}.

XGBoost (eXtreme Gradient Boosting) is an efficient and flexible machine learning model based on gradient boosting. The optimized parameters include the learning rate (learning_rate), maximum tree depth (max_depth), subsample ratio (‘subsample’), and regularization coefficient (reg_lambda). The optimization method is Bayesian Optimization combined with a 5-fold cross-validation, and the optimal parameters for the final: {‘learning_rate’: 0.1, ‘max_depth’: 5, ‘subsample’: 0.8, ‘reg_lambda’: 1}.

CNN (Convolutional Neural Network): The optimized parameters include the number of convolutional layers (layers), number of filters (‘filters’), learning rate (learning_rate), and dropout rate (dropout_rate). The optimization method is Random Search combined with early stopping on the validation set, and the optimal parameters for the final: {layers: 3, ‘filters’: 64, ‘learning_rate’: 1 × 10^−3^, ‘dropout_rate’: 0.5}.

XGraphBoost method integrates GNNs with the XGBoost algorithm for classification tasks. The optimized parameters include the learning rate (learning_rate), maximum tree depth (max_depth), graph embedding dimension (graph_embedding_dim), and number of graph attention heads (num_heads). The optimization method is Random Search combined with a 3-fold cross-validation, and the optimal parameters for the final: {‘learning_rate’: 0.1, ‘max_depth’: 5, ‘graph_embedding_dim’: 32, ‘num_heads’: 2}.

FraGAT, based on GNN: The optimized parameters include the number of attention heads (heads), the hidden layer dimension, the dropout rate (hidden_dim), and the learning rate (learning_rate). The optimization method is Grid Search combined with early stopping on the validation set, and the optimal parameters for the final: {‘heads’: 4, ‘hidden_dim’: 128, ‘dropout’: 0.5, ‘learning_rate’: 1 × 10^−4^}.

The comparative experiments analyzed the results using seven metrics: Accuracy, Sensitivity and Specificity, MCC, F1-score, PRC, and AUC. Sensitivity and Specificity quantify the ability to recognize positive and negative classes, respectively. Given the class imbalance in the dataset (2:3 ratio), we used the MCC and F1-score to assess the overall balance. PRC and AUC were employed to verify threshold-independent robustness, addressing the limitations of accuracy in class imbalance scenarios comprehensively. The comparison results are presented in Table 1.

Notably, we divided the training, validation, and test sets using 10 different random seeds to ensure a diverse sampling. Subsequently, the model was assessed using a 10-fold cross-validation to guarantee robust results. For each random seed, we documented the model’s average performance on the validation or test set and selected partitions that exhibited the best overall performance across multiple metrics. In the selection process, we prioritized the Acc metric, with the MCC and F1-score being secondary considerations, leading to the identification of the following optimal set of outcomes.

As shown in Table 1, it can be observed that our model outperformed the other seven algorithms across all of the metrics: the test Accuracy, Sensitivity, Specificity, MCC, F1-score, PRC, and AUC. This indicated that the model had significant advantages in the overall classification performance, class balance (MCC), precision-recall trade-off (F1-score), and robustness with imbalanced data (PRC). This demonstrates that the proposed model, which integrates molecular graph structures and SMILES sequences, including feature selection, network architecture, and loss functions, contributed to a substantial performance improvement.

A high sensitivity of 85.2% and specificity of 89.4% indicated that the model had a very strong ability to identify both positive and negative class samples. The high MCC (0.744) suggested that the model demonstrates an excellent overall performance even when dealing with class imbalance, avoiding the limitations of relying solely on a single metric, such as accuracy. An F1-score of 0.872 indicated that the model achieved a good balance between precision and recall. It reflected the model’s effectiveness in correctly identifying positive instances while minimizing false positives and false negatives. Even more to the point, the simultaneous improvement in the MCC and F1-score revealed the synergistic optimization of model predictions. The former reflected the overall coordination of the contingency table (TP/TN/FP/FN), while the latter confirmed an enhanced precision-recall trade-off. This concurrent optimization was particularly critical in scenarios of class imbalance. As noted by Chicco et al. [24], when the class ratio deviates from 1:1 (e.g., 2:3), MCC provides more valuable insight than individual metrics. In addition, the improvement of PRC indicated that the model maintains a good balance between the Accuracy and F1-score under different thresholds, and can effectively cope with different application scenarios.

We have also observed that the model’s training accuracy (94.1%) was significantly higher than its validation (86.5%, which is also calculated but not included in Table 1) and test set accuracy (87.7%), indicating a potential overfitting. We attributed this to the model’s inherent complexity combined with a relatively small training dataset, where excessive model parameters might have led to an over-specialization on the training data. In future work, we aim to acquire more training data to mitigate this issue and plan to further optimize the model by adopting regularization strategies, such as enhancing Dropout, applying weight decay (L2 regularization), or exploring data augmentation techniques to improve the generalization capability.

Overall, the proposed fusion learning model demonstrated significant performance advantages on the training set, achieving an accuracy of 0.941, which represented improvements of 4.6%, 10.2%, and 14.4% over current mainstream deep learning benchmark models FraGAT (0.895), XGraphBoost (0.839), and CNN (0.797), respectively. Compared to the classical RF method [7] (0.850), the accuracy improvement reached 9.1%, showcasing a more competitive feature representation capability. It is worth noting that although traditional machine learning methods such as SVM (0.993), KNN (0.994), and XGBoost (0.961) exhibited higher values on the training set, their performance on the test set indicated a potential sensitivity to local features of the training data, implying a risk of overfitting. In contrast, our model achieved an optimal value of 0.877 on the test set, demonstrating a more robust generalization balance. This indicated that our model could capture the essential patterns of the data through deep feature learning while avoiding overfitting, thereby possessing a greater practical application potential in complex tasks.

**Figure 1:**
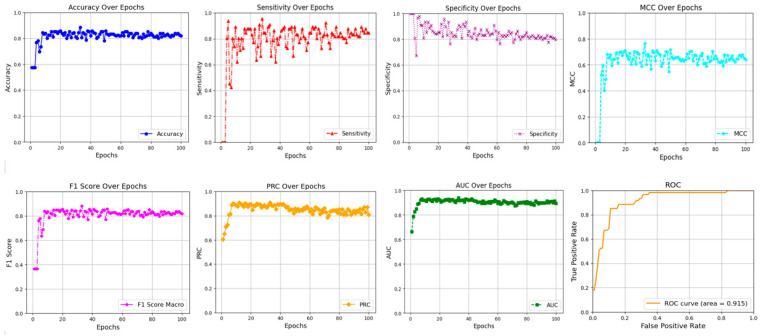
The performance of the metrics in the GSFL model on the BACE-1 dataset.

To better illustrate the training process of the proposed model, we plotted the experimental progress curves on the BACE-1 dataset, as shown in Figure 1. The *x*-axis represents the number of iterations, while the *y*-axis denotes the performance of the metrics, with each metric depicted by a distinct colored curve. This figure visualized the variations of the seven classification metrics (listed in Table 1) across training iterations, alongside the ROC curve (with an area under the curve of 0.915), demonstrating the excellent classification performance of our proposed model.

In future work, we plan to develop an adjustable model framework tailored to different application scenarios to meet various performance requirements. In summary, the method proposed in this study excels in several key performance metrics, demonstrating its potential for practical application. We will continue to optimize the model to achieve more balanced and comprehensive performance improvements.

### 2.3. Ablation and Discussion

This experiment conducted ablation studies to evaluate the contribution of key modules or algorithms, including graph models, a sequence model, atomic-level Feature Attention (FA), a Hierarchical Attention (HA) mechanism, the Functional Group Splitting (Fg_Splitting) method, and their fusion experiments. By comparing the results with and without these components, we analyzed their specific impact on model performance.

**Figure 2:**
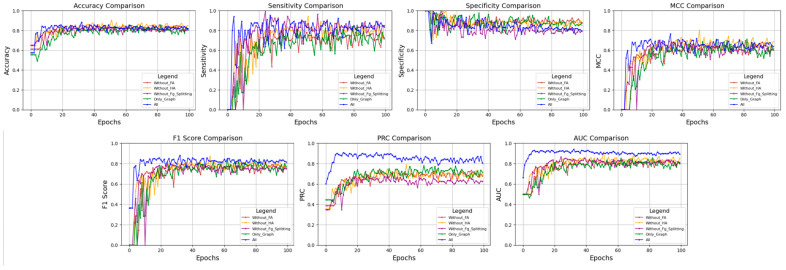
A performance comparison of the ablation experiments for the classification prediction task.

Firstly, we presented the performance of the model using GNN with an FA module to demonstrate the performance improvement achieved by incorporating the Sequence model. Next, we evaluated the FA mechanism and analyzed its specific impact on model performance. Following that, we assessed the HA mechanism and examined its influence on model performance. Finally, we evaluated the impact of the Fg_Splitting module used for pre-processing the sequence model on the overall model performance. The experimental results were summarized in Table 2 and visualized in Figure 2, where the horizontal axis represented the training epochs, while the vertical axis showed the changes in various metrics.

As shown in Table 2, the complete model (All) surpassed other ablated models across key performance metrics, with notable enhancements in Accuracy (0.877), Sensitivity (0.852), MCC (0.744), F1 score (0.872), PRC (0.869), and AUC (0.915). By contrast, the model without the FA mechanism exhibited a decreased performance in MCC (0.698), F1-score (0.810), and PRC (0.741), along with a slightly lower AUC (0.899). The model lacking the HA mechanism also underperformed the complete model in MCC (0.716), F1-score (0.814), and PRC (0.735), with a somewhat inferior AUC (0.895). Moreover, the model without Fg_Splitting demonstrated a decline in MCC (0.685), F1-score (0.792), and PRC (0.706), and its AUC (0.881) is significantly lower than that of the complete model. The graph-only model, despite achieving comparable results to the complete model in MCC (0.712), F1-score (0.838), PRC (0.780), and AUC (0.909), still had room for improvement in Accuracy (0.858) and Sensitivity (0.826).

In summary, the integration of FA, HA, and Fg_Splitting mechanisms is vital for boosting the model’s classification performance, class balance, precision-recall trade-off, and robustness in dealing with imbalanced data. The complete model’s superiority over other ablated models across multiple key metrics indicates its better comprehensive performance and robustness in classification tasks. The ablation study reveals that removing the HA mechanism leads to a drop in performance in MCC, F1-score, and PRC, although AUC sees a slight increase, which confirms the crucial role of the HA mechanism in enhancing the model’s overall performance. Additionally, the model without Fg_Splitting shows a decreased performance in MCC, F1-score, and PRC, highlighting the significance of integrating chemical knowledge to improve classification performance. The ablation study of the graph-only model suggests that while the graph model can capture molecular structure information, its overall performance still needs improvement when making complex decisions based on multiple types of features.

Based on the results of the ablation experiments, we can consider making improvements in the future: Firstly, analyzing misclassified samples may reveal the reasons for the decline in specificity and suggest new optimization directions, such as optimizing specificity by adjusting the model architecture or introducing new regularization methods to balance sensitivity and specificity. Secondly, adopting multi-objective optimization in the dataset strategy to achieve a better balance among Sensitivity, Accuracy, Specificity, and AUC, could be realized by adjusting the loss function or introducing weighted strategies. Thirdly, we could use data augmentation techniques to expand the dataset, thereby improving the model’s generalization ability and robustness, which may enhance performance across various metrics. Fourthly, further fine-tuning the FA module by conducting experiments to study the impact of its internal parameters on model performance, in order to assess the effects of these parameter settings on specificity could occur. Lastly, we can consider the introduction of additional features or using feature selection techniques to enhance the model’s predictive capability, as new features may provide more information that helps improve specificity and other metrics.

## 3. Materials and Methods

### 3.1. Dataset

The bioactivity dataset, comprising 1548 BACE-1 inhibitors, was sourced from the study reported by Bao et al. [7], which was originally extracted from the ChEMBL database (available at https://www.ebi.ac.uk/chembl/ accessed on 10 April 2022). Detailed information regarding the dataset is provided in Supplementary Table S1. All compounds were classified under the organism Homo sapiens, with a focus on a single target protein. To ensure consistency, the half-maximal inhibitory concentration (IC50) values were converted to nanomolar (nM) units. Following this, a data cleaning process was implemented, which involved the elimination of duplicate chemical structures, compounds exhibiting ambiguous inhibitory activity (i.e., inconclusive IC50 values), and salts. Furthermore, in line with the validity criteria specified in the ChEMBL database, compounds identified as “outside typical range” or flagged for “potential transcription error” were excluded from the dataset.

In the dataset, binary classification was performed based on these logIC50 values: 618 molecules with logIC50 values less than 2 were labeled as having inhibitory activity, while 930 molecules with logIC50 value greater than 2 were labeled as lacking inhibitory activity, resulting in an approximately 2:3 ratio. For the purposes of this experiment, the dataset was randomly divided into training, validation, and test sets in an 8:1:1 ratio.

### 3.2. Methods

**Figure 3:**
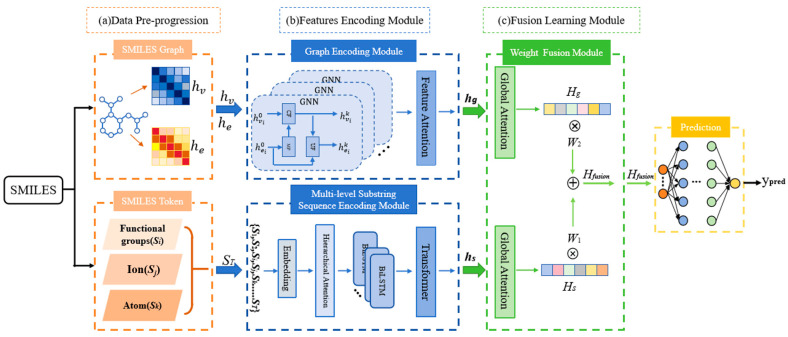
Overview of the proposed method. (**a**) Data pre-progression: the converting of SMILES strings to graph data and multi-level substrings from SIMILES; (**b**) feature encoding module: the graph encoding model with an atomic-level feature attention mechanism and the BiLSTM–Transformer framework with a hierarchical attention mechanism; and (**c**) the fusion learning module.

Figure 3 provided an overview of the GSFL model. The framework comprised four key modules: data pre-processing, molecular graph encoding, multi-level substring sequence encoding, and fusion learning. During the data pre-processing stage, as shown in the Figure 3a stage, we utilized RDKit [25] and PyTorch Geometric (PyG) libraries [26] for SMILES string parsing and molecular graph construction. The former is an open-source cheminformatics toolkit extensively employed in chemical structure processing and analysis. It provides a robust functionality to efficiently parse SMILES strings and convert them into molecular graphs with atom/bond attributes. The latter serves as a specialized GNN library built upon PyTorch, which enables the efficient construction and training of graph-based models for molecular data processing. This integration facilitates a comprehensive feature extraction spanning functional groups, ionic states, and atomic-level characteristics while preserving molecular topology. The molecular graph encoding module employed multiple GNNs enhanced with an atomic-level attention mechanism, as depicted in the Figure 3b stage. For multi-level substring sequence encoding, we implemented a BiLSTM framework equipped with a hierarchical attention mechanism, as depicted also in the Figure 3b stage. The fusion learning module concatenates feature vectors after global pooling, illustrated in Figure 3c.

#### 3.2.1. Data Pre-Processing

(1). Molecular Graph Construction

The experimental data from the ChEMBL database consisted of 1548 compounds, each with BACE-1 activity measurements, all presented in a SMILES (Simplified Molecular Input Line Entry System) format. SMILES is a simplified text format used to represent the structure of chemical molecules, commonly used in cheminformatics and drug design. We utilized RDKit to parse SMILES strings and convert them into molecular objects. These objects were then developed into molecular graphs, which were converted into graph data for use as inputs in graph encoding models. However, when using SMILES for molecular representation, it is unable to directly convey stereochemical information, and there is a non-uniqueness in representing complex molecular structures. These issues require additional information or algorithms to supplement or resolve them.

**Figure 4:**
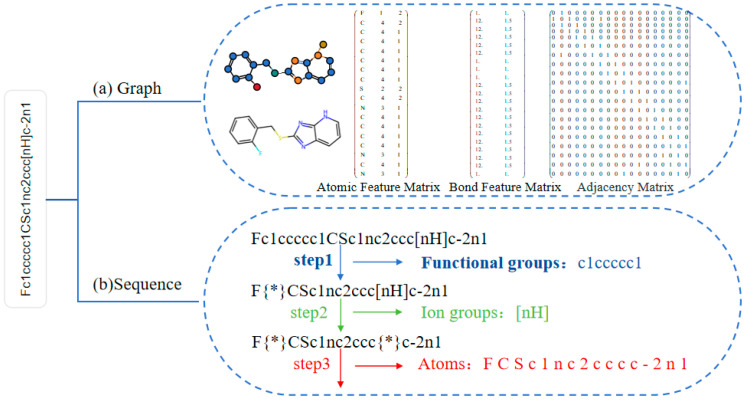
Representations of converting SMILES string into graphs and multi-level substrings. There “{*}” represents the extracted substrings.

The graph data of a molecular graph is determined by the features of atoms (corresponding to graph nodes) and chemical bonds (corresponding to graph edges), where the feature dimensions are predefined by specific chemical properties. Each property is represented either through one-hot encoding or scalar values, with a fixed number of attribute categories. For example, atom features include atom type, degree, charge, chirality, number of hydrogen atoms, hybridization, aromaticity, and atomic mass, while bond features include bond type, conjugation, ring membership, and stereochemistry. After transformation, the feature dimensions for atoms and bonds are 133 and 14, respectively. Consequently, the atom feature matrix has dimensions [number of atoms, 133], and the bond feature matrix has dimensions [number of bonds, 14]. For instance, for the SMILES formula “c1(ccc2nc3CCCCc3c (c2c1)N)N”, the graph node feature matrix shape is [16, 133] and the graph edge feature matrix shape is [18, 14]. The diagram illustrating the conversion process from a molecular graph to graph data is shown in Figure 4a.

(2). Multi-level substring sequences extraction

Currently, some sequence models input atom-level elements and chemical bonds from SMILES directly for training. However, this approach often overlooked important substructure information within the molecule. Ren et al. [27] proposed a substructural hierarchical attention network that provided a multi-perspective view of the molecular. In addition, traditional atom-level annotation methods have certain limitations, primarily manifested in the ambiguity of representing the same ion in different character forms. To tackle this issue, our research has taken note of the Functional Group BERT (FG–BERT) [28] strategy, which improved molecular property prediction by focusing on informative features from functional groups. We have meticulously analyzed SMILES molecules’ substructures, particularly functional groups with distinct chemical traits. Following SMILES rules, we have also broken down other molecular segments into free-state ion groups, ensuring a thorough examination alongside all of the remaining atoms and bonds.

Specific practices were as follows: Firstly, we extracted high-frequency SMILES functional group vocabularies from the BACE-1 dataset and annotated them with SMILES notation, enhancing the readability and chemical interpretability of these functional groups to better support the training of deep learning models. Next, based on the SMILES encoding rules, we extracted the structures between the “[“and”]” symbols in the remaining parts of the molecule, treating them as ion groups for processing. For example, the stereochemical encoding “[C@@H]” contained rich spatial structural information but was split into six independent characters. Therefore, we treated these special substrings (e.g., “[nH]”, “[O-]”, “[C@]”, etc.) as a whole during processing. Finally, for the remaining parts of the molecule, each character was separately parsed to extract atomic and branching bond structural features, with each extracted or parsed element being treated as a token in the SMILES sequence and encoded individually. The structural representations of functional groups, ion groups, and atoms in SMILES are shown in Table 3. Functional groups, ion groups, and atomic structure decomposition in a specific SMILES example are shown in Table 4. The process of splitting the SMILES is shown in Figure 4b.

#### 3.2.2. Graph Encoding Module

**Figure 5:**
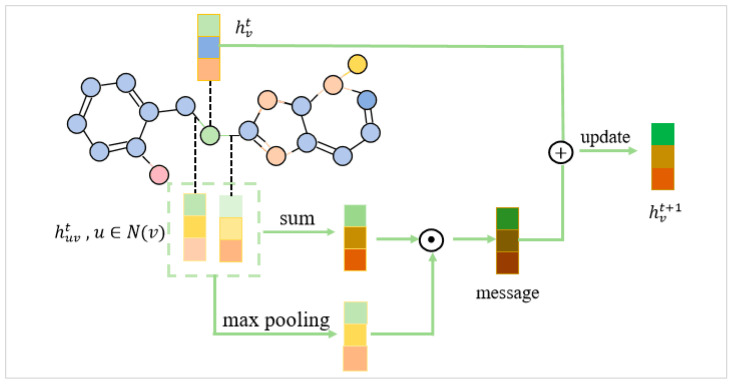
The graph neural network with a message passing mechanism.

A molecule is made up of atoms connected by chemical bonds. The molecule can be represented as a graph $G=\left(V,E\right)$, with V denoting the set of atoms and E denoting the set of chemical bonds. The encoder feeds the node and edge feature matrices corresponding to the atoms and bonds of a molecule into the GNNs, which iteratively refines these features to capture crucial chemical information from the molecule. The encoder passes the extracted atomic and chemical bond features into a GNN module consisting of multiple communication message passing mechanisms [29,30]. Then, the features were fed into an atomic-level attention mechanism to construct an interatomic interaction layer, enabling the model to better capture local atomic interactions and emphasize key information within the molecule. The feature transmission process of the graph neural network with a message passing mechanism was shown in Figure 5.

(1). Message Passing with an Atomic-Level Feature Attention Encoder

The basic graph encoder was used to obtain atomic representations and consists of several GNN layers. Each GNN layer captured the interactions between atoms in the molecule.

Next, the basic GNN layer was applied, where graph features were initialized using separate linear layers to map the input atomic and bond features to a hidden space. When processing the initial features of atoms, this model primarily considers atomic types (such as C, H, and O), valence electrons, and hybridization states, which directly characterize the bonding capacity and geometric structure of atoms. For bond features, the model relies on bond types and geometric details to reflect the connection strength between atoms and the stability of molecules. These initial features are ultimately mapped to hidden states through linear layers, generating initial hidden vectors for atoms and bonds, resulting in initial hidden vector dimensions of [number of atoms, 133] for atoms and [number of bonds, 14] for bonds. The input atomic feature matrix $X_{V}\in R^{N_{V}\times d_{V}}$ and chemical bond feature matrix $X_{E}\in R^{N_{E}\times d_{E}}$ was mapped to the hidden space via a linear transformation matrix $W_{i}^{V}\in R^{d_{V}\times d_{hidden}}$ and $W_{i}^{E}\in R^{d_{E}\times d_{hidden}}$, yielding the initial representation for each atom and chemical bond:

(1) $$h_{v}^{0}=ReLU\left(X_{V}W_{i}^{V}\right)$$

(2) $$h_{e}^{0}=ReLU\left(X_{E}W_{i}^{E}\right)$$

where $d_{V}$, $d_{E}$, and $d_{hidden}$ denoted the dimensionalities of the atom features, chemical bond features, and hidden space, respectively. $N_{V}$ and $N_{E}$ represented the number of atoms and chemical bonds, respectively. *ReLU* was the non-linear activation function applied element-wise. The feature representations $h_{v}^{0}$ and $h_{e}^{0}$, processed through linear transformations and activation functions, will serve as inputs for information propagation and aggregation in subsequent layers.

The core of the GNN layer lied in how to effectively utilize the atomic and chemical bond information from the raw molecular graph structure to extract meaningful features through aggregation and updating processes. The input was a graph, $G=\left(V,E\right)$, including node attributes $X_{V}$ and edge attributes $X_{E}$. Initial node hidden representation was denoted as $h_{v}^{0}=x_{v}$, and the hidden representation of the edge was denoted as $h_{e}^{0}=x_{e}$. They propagated in the following way during the $\bar{k}$ iteration:

(1) Aggregation:
  (3) $$m_{v}^{k}=AGGREGATE\left(h_{e}^{k-1}\right)$$
  where $m_{v}^{k}$ was the message generated by the node.
(2) Communication:
  (4) $$h_{v}^{k}=COMMUNICATE\left(m_{v}^{k},h_{v}^{k-1}\right)$$
  where $h_{v}^{k}$ was the hidden representation of the node.
(3) Edge representation update:
  (5) $$h_{e}^{k}=\sigma \left(h_{e}^{0}+W\cdot \left(h_{v}^{k}-h_{e}^{k-1}\right)\right)$$
  where $h_{v}^{k}$ represented the hidden representation of the node, and $h_{e}^{k}$ represented the hidden representation of the edge. Here, W was a learnable weight matrix, and the activation function was denoted as σ. After L iterations, the following operations were performed to obtain the final message and node representation.
(4) Final message aggregation:
  (6) $$m_{v}=AGGREGATE\left(h_{e}^{L}\right)$$
(5) Final node representation update:
  (7) $$h_{v}=COMMUNICATE\left(m_{v},h_{v}^{L},x_{v}\right)$$

The AGGREGATE function included a message enhancer that generated the maximum pooling result of the sum of the edge hidden states *he* and computed the element-wise product of the sum of *he*. The COMMUNICATE(·) represented a multilayer perceptron (MLP). This process enhanced atomic-level information exchange by focusing on the most relevant connections in the graph structure, optimizing node representations using message aggregation and attention mechanisms.

(2). Feature Attention Module

**Figure 6:**
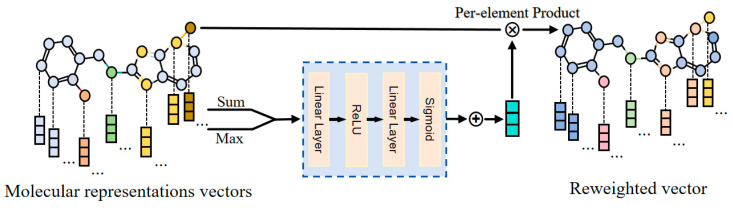
The detailed structure of the Feature Attention Module.

Herein, the Feature Attention (FA) module leveraged the molecular feature representation vector to adaptively focus on the feature dimensions of each atom within the same molecule, re-evaluated and adjusted the weights of the atomic feature vectors, and ultimately generated the reconstructed feature vector. The detailed information is listed in Figure 6.

Once the updated hidden state $h_{v}$ was obtained, we first condensed the temporary global information into two distinct feature descriptors:

(8) $$g_{sum}=\sum_{v=1}^{N}h_{v}$$

(9) $$g_{max}=max\{h_{v}|v\in V\}$$

which represented sum pooling and max pooling across different atomic feature channels in the molecule, respectively.

Then, we inputted $g_{sum}$ and $g_{max}$ into a shared excitation operator to obtain the feature attention weights c∈ $R^{d}$ as follows:

(10) $$c=Sigmoid\left(W_{1}(Relu\left(g_{sum}\cdot W_{2}\right)\;+Relu\left({g_{max}\cdot W}_{2}\right))\right)$$

where $W_{1}\in R^{\left(d/r\right)\times d}$ and $W_{2}\in R^{d\times \left(d/r\right)}$ were trainable parameters, and Sigmoid and Relu referred to the non-linear activation function, respectively. The reduction ratio r was utilized to limit model complexity and aid generalization [31]. Therefore, $h_{v}^{\prime }$ was reweighted according to the following:

(11) $$h_{v}^{\prime }=c⊙h_{v}$$

where ⊙ was the element-wise product and $h_{g}$ was the reweighted hidden state. Consequently, a set of reweighted hidden states $h_{g}=\{h_{1}^{\prime },h_{2}^{\prime },...,h_{v}^{\prime },...,h_{V}^{\prime }\}$ was sent to the next layer.

#### 3.2.3. Multi-Level Substring Sequence Encoding Module

**Figure 7:**
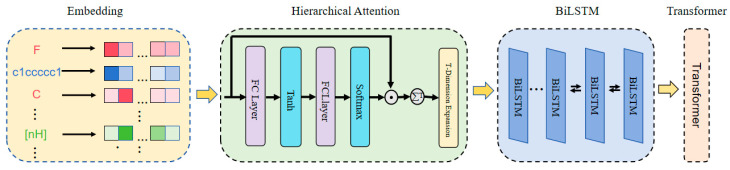
Multi-level substring sequence encoding module.

As described above, we converted BACE-1 inhibitor molecules into three levels of sequence information, including functional group, ion, and atom level. In this module, a hierarchical attention mechanism was designed to enable a layer-wise weighted learning of features at different levels, allowing the model to adaptively assign attention weights between features at different hierarchies, thereby effectively extracting the detailed information at each level. The details of the multi-level substring sequence encoding module are illustrated in Figure 7 and mainly contained SMILES embedding, hierarchical attention, and BiLSTM and Transformer architectures coding steps [32].

(1). SMILES Embedding

A SMILES molecular sequence is composed of multiple subsequences $S=\{s_{i}|i=1,\cdots ,s_{T}\},s_{i}\in D$, where each token $s_{i}$ is derived from three different levels of subsequences, and T represents the number of times the SMILES sequence is split. D represents the total dictionary constructed from the token subsequences. For each SMILES molecular sequence, we used one-hot encoding to map the sequence to a feature vector $X_{s}$ = {$x_{s_{i}}|i=$1, ⋯, *t*}, $x_{s_{i}\;}\in R^{\left|D\right|}$.

(2). Hierarchical Attention

Subsequently, we referenced the hierarchical attention network proposed by Yang et al. [32] and developed a functional module capable of effectively distinguishing between SMILES molecular sequences and their subsequences. Following this, we provided a detailed description of the implementation of the Hierarchical Attention (HA) module. The detailed information of the HA module is shown in Figure 7. In this module, a linear transformation was applied to the input features, where the input consisted of subsequence embedding vectors, denoted as $X\in R^{B\times T\times H}$, with B representing the batch size, T the sequence length, and H the dimensionality of the input features. The linear transformation was defined as follows:

(12) $$Z=tanh\left(W_{1}\cdot X+b\right)\in R^{B\times T\times A}$$

Here, $W_{1}\in R^{A\times H}$ was the weight matrix, $b\in R^{A}$ was the bias term, and A was the dimensionality of the attention features. The *tanh* was a hyperbolic tangent activation function. The context vector was computed by applying a linear transformation to the output:

(13) $$S=SM\left(W_{2}\cdot Z\right)\in R^{B\times T\times 1}$$

Here, $W_{2}\in R^{A\times 1}$ was the weight matrix and SM denoted the Softmax function, which was used to normalize the scores. By computing the weighted value for each input:

(14) $$X_{atten}=X⊙S\in R^{B\times T\times H}$$

Here, the ⊙ representation was element-wise multiplication.

By aggregating the weighted input and combining it with the original input:

(15) $$h_{atten}=\sum_{t=1}^{T}X_{atten}\in R^{B\times H}$$

The variable was expanded along the T dimension, resulting in the updated output:

(16) $$h^{\prime }=Expand(h_{atten},T)$$

The final output shape $h^{\prime }$ was B×T×H.

The HA module dynamically weighted the encoding vectors of different levels (atomic, ionic, and functional group) using attention weights to calculate feature relevance, effectively fusing multi-level features and capturing contextual information. This mechanism integrated local information into a global molecular representation, enhancing the learning performance of the subsequent BiLSTM model and improving the expressive power of molecular features.

(3). BiLSTM and Transformer Coding Module

Introducing a hierarchical attention mechanism before feeding a sequence into a BiLSTM can help focus on key substructures within molecular sequences. By progressively concentrating on different layers or granularities, from molecular functional groups to ion groups and then to atoms, the mechanism learns attention weights at each level. This approach effectively captures important features and contextual information, enabling the model to grasp both global insights and adaptively adjust its handling of various substructures based on the specific task. Consequently, this significantly enhances prediction accuracy.

BiLSTM, as an enhanced recurrent neural network, can simultaneously utilize both the forward and backward information of the BACE-1 inhibitor molecular sequence. Due to the non-directional nature of SMILES, a unidirectional recurrent neural network cannot accurately capture molecular information. It consisted of two independent LSTM networks, each used to process the forward and backward information of the encoded sequence. Specifically, for the input of the BACE-1 SMILES sequence $X_{s}=\{x_{s_{i}}|i=1,\cdots ,t\},\;x_{s_{i}}\in R^{\left|D\right|}$, the output of the BiLSTM can be represented as follows:

(17) $$\left\{\begin{array}{l} \vec{h_{s_{i}}}=\vec{LSTM}\left(x_{s_{i}},\vec{h_{s_{i-1}}}\right)\\ \overset{\leftarrow }{h_{s_{i}}}=\overset{\leftarrow }{LSTM}\left(x_{s_{i}},\overset{\leftarrow }{h_{s_{i-1}}}\right) \end{array}\right.$$

Here, $\vec{h_{s_{i}}}$ represented the forward $s_{i}$ hidden state, $\overset{\leftarrow }{h_{s_{i}}}$ represented the backward $s_{i}$ hidden state, and $x_{s_{i}}$ was the $s_{i}$ input subsequence in the input sequence. The final output of the BiLSTM combined both the forward and backward hidden states, and was represented as follows:

(18) $$h_{s_{i}}=\vec{h_{s_{i}}}⊕\overset{\leftarrow }{h_{s_{i}}}$$

The operation ⊕ represented concatenation.

After BiLSTM performed the bidirectional encoding of the SMILES sequence, the result was passed to the Transformer. The Transformer used self-attention mechanisms to extract the latent representation of the SMILES sequence.

#### 3.2.4. Weight Fusion Learning Module

In order to fuse the information of different modes effectively, by leveraging the GLOBAL Attention mechanism in the PyTorch Geometric deep learning framework, we integrated the different modality representations of each atom within a molecule to obtain the graph-level structural representation of the entire SMILES molecule, thus deriving the unified feature vectors $H_{s}$ and $H_{g}$ corresponding to the two modalities. Utilizing this global feature layer, the embedding vectors of each atom’s features within a molecule were fused into a unified graph representation of the entire SMILES structure, which allowed for the extraction of the corresponding unified feature vectors $H_{s}$ and $H_{g}$ under the two modalities. The detailed process is demonstrated in part (c) of Figure 3.

Subsequently, the two feature vectors *Hs* and *Hg*, with unified dimensionality, were passed into a weight fusion layer that we designed to generate a comprehensive representation of the molecule. That is, to generate a comprehensive representation layer, which combined the features of different modes weighted by nonlinear functions, and added a bias term. The formula was as follows:

(19) $$H={f(H}_{s},H_{g})+b=W_{s}\cdot H_{s}+W_{g}\cdot H_{g}+b$$

where $H_{s},\;H_{g}$ represented the feature representation of sequence-based and graph-based neural network methods, respectively, $W_{s},\;W_{g}$ represented the learnable weight, and b represented the bias term.

In the end, the fully connected prediction model consists of three layers: an input layer (fusion_dim × 256), a hidden layer (256 × 64), and an output layer (64 × 1). Between the input and hidden layers, we incorporated a ReLU activation function followed by a dropout regularization to mitigate overfitting. The hidden and output layers are connected via another ReLU activation. This architecture balances model capacity and generalization, aligning with common practices in deep learning for molecular property prediction.

#### 3.2.5. Loss Function

In molecular property prediction tasks, it is crucial to effectively represent the feature vectors of two modalities and integrate these features. The differences in feature representation across modalities need to be addressed. To effectively reduce the discrepancies in feature representations from different methods, we proposed a loss function based on similarity computation. This method effectively minimized the differences between different representations, thus facilitating a better fusion of the two modalities’ features.

Fusion Contrastive Loss:

(20) $$L_{Contrast}=\sum_{r\in \{g,s\}}\left(-\frac{1}{N}\sum_{i=1}^{N}log\frac{exp\left(\frac{cos\left(Z_{s}\left(i\right),Z_{g}\left(i\right)\right)}{T}\right)}{\sum_{j=1,j\neq i}^{N}exp\left(\frac{cos\left(Z_{s}\left(i\right),Z_{g}\left(j\right)\right)}{T}\right)}\right)$$

Here, $Z_{g}\left(i\right)$ and $Z_{s}\left(i\right)$ represented the feature vectors of the *i*-th sample g and the sample s, respectively, while cos(·) denoted the cosine similarity. T represented the contrastive loss ratio, which N corresponded to the quantity of sample information. r ∈ {g, s} served as an identifier for the feature space, where r can take the values g or s, representing the graph structure feature space and the sequence information feature space, respectively. By incorporating the contrastive loss, the model’s ability to distinguish between differences in feature vectors was enhanced, while the inclusion of the label loss ensured the accuracy of the model’s predictions.

In classification prediction tasks, binary cross-entropy loss (BCEWithLogitsLoss) was used, combined with contrastive loss.

(21) $$L_{Fusion\_Contrast}=L_{BCE}+\alpha L_{Contrast}$$

Among them, binary cross-entropy loss (BCELoss) was as follows:

(22) $$L_{BCE}=\sum_{i=1}^{N}BCEWithLogitsLoss(pred_{i},label_{i})$$

where α was a hyperparameter that differed in magnitude between the contrastive loss and the prediction task loss, used to balance the label loss and the contrastive loss, making the training more stable.

### 3.3. Metrics

In this study, seven metrics were used to evaluate the classification model: Accuracy (ACC), Sensitivity (SE), Specificity (SP), Matthews correlation coefficient (MCC), F1-score, Precision–Recall Curve (PRC), Area Under Curve (AUC), and the Acc is the main basis of model tuning. These metrics are defined below as follows:

(23) $$Accuracy=\frac{TN+TP}{TN+FP+TP+FN}$$

(24) $$SE=\frac{TP}{TP+FN}$$

(25) $$SP=\frac{TN}{TN+FP}$$

(26) $$MCC=\frac{TP\times TN-FP\times FN}{\sqrt{\left(TP+FP\right)\left(TP+FN\right)\left(TN+FP\right)\left(TN+FN\right)}}$$

(27) $$F1-score=\frac{2\times TP}{2\times TP+FP+FN}$$

(28) $$TPR=\frac{TP}{TP+FN},\;\;\;FPR=\frac{FP}{FP+TN}$$

where TP is the True Positive; FP is the False Positive; TN is the True Negative; and FN is the False Negative. Accuracy reflected the proportion of correct predictions out of the total samples. AUC evaluated the model’s performance, with the True Positive Rate (TPR) and False Positive Rate (FPR) used to plot the ROC curve. The PRC showed the relationship between precision and recall, with a larger area indicating a better model performance. Sensitivity (SE) was the proportion of actual positive samples correctly predicted as positive, and Specificity (SP) was the proportion of actual negative samples correctly predicted as negative. The F1-score provided a balanced evaluation of precision and recall, with a maximum value of 1; higher values indicated a better model. The Matthews Correlation Coefficient (MCC) ranged from −1 to 1 and reflects predictive performance, which is particularly effective in cases of imbalanced samples.

## 4. Conclusions

The development of BACE-1 inhibitors remains a critical focus in the pursuit of early AD treatment. Traditional GNNs often overlook explicit sequence-level semantic information when predicting molecular activity. To address this limitation, we have developed a novel graph and sequence fusion learning model specifically designed for predicting the molecular activity of BACE-1 inhibitors. Our approach uniquely extracted frequently occurring functional groups from the dataset to create distinct sequence markers based on SMILES coding rules. These markers were then combined with ion and atomic level substrings and encoded using a BiLSTM–Transformer framework enhanced by a hierarchical attention mechanism. Compared to other leading methods, our model demonstrated a superior reliability across multiple metrics. These results underscored the model’s effectiveness in accurately predicting inhibitory activity, highlighting its potential advantages in the field. However, it is essential to acknowledge certain limitations of our study. The computational complexity of our model may pose challenges in terms of scalability and processing time, particularly when applied to larger datasets. Additionally, potential biases in the dataset could affect the generalizability of our results. Future work should focus on addressing these issues, exploring more efficient algorithms, and validating our findings across diverse datasets to enhance the robustness and applicability of our model in real-world scenarios.

## Figures and Tables

**Table 1:** A comparison of the related models in the experimental results. There the upward arrow indicates that higher values are better.

Methods	Accuracy ↑		SE ↑	SP ↑	MCC ↑	F1-Score ↑	PRC ↑	AUC ↑
	Training	Test						
SVM	0.993	0.810	0.694	0.887	0.598	0.745	0.680	0.790
KNN	**0.994**	0.813	0.758	0.849	0.609	0.764	0.681	0.804
XGBoost	0.961	0.816	0.734	0.871	0.613	0.762	0.687	0.802
CNN	0.797	0.797	0.815	0.785	0.590	0.762	0.658	0.799
RF [7]	0.850	0.830	0.850	null	0.660	0.800	null	0.880
XGraphBoost [11]	0.839	0.834	0.814	0.855	0.667	0.813	0.857	0.891
FraGAT [13]	0.895	0.857	0.825	0.876	0.696	0.810	0.833	0.911
Ours	0.941	**0.877**	**0.852**	**0.894**	**0.744**	**0.872**	**0.869**	**0.915**

**Table 2:** Ablation experiment metrics for the classification task. There the upward arrow indicates that higher values are better.

Metrics	Accuracy ↑	SE ↑	SP ↑	MCC ↑	F1-Score ↑	PRC ↑	AUC ↑
Without FA: Graph + Sequence + HA + Fg_Spliting	0.858	0.783	0.905	0.698	0.810	0.741	0.899
Without HA: Graph + Sequence + FA + Fg_Spliting	0.871	0.800	**0.910**	0.716	0.814	0.735	0.895
Without Fg_Spliting: Graph + Sequence + HA + FA	0.858	0.778	0.901	0.685	0.792	0.706	0.881
Only Graph model: Graph + FA	0.858	0.826	0.884	0.712	0.838	0.780	0.909
All	**0.877**	**0.852**	0.894	**0.744**	**0.872**	**0.869**	**0.915**

**Table 3:** The structural representations of functional groups, ionic groups, and atoms.

Functional Groups	Ion Groups	Atoms
C(=O)N, OCC, [N+](=O)[O−], clccccc1 S(=O), O=c, NC(=O), NS(=O)(=O), OC, S(=O)(=O), C=O, O=C, [N+](=O)[O−]	[C@H], [C@@H], [C@@], [nH], [C@], [S@+], [S+], [N+], [S@@+], [N+](=O)[O–], [O]	C, c, O, N, S, F, Cl, Br, I, =, –, #, /, \, 1, 2, 3, 4, 5, 6

**Table 4:** Functional groups, ion groups, and atomic structure decomposition in a specific SMILES example.

SMILES	Substructures		
	Functional Groups	Ion Groups	Atoms
CC(C)CNC(=O)[C@H](C)[C@H](O)[C@H](CC(C)C)NC(=O)[C@H]C(Cc1ccccc1)NC(=O)c1ccccc1	NC(=O), NC(=O) Cc1ccccc1, NC(=O), c1ccccc1	[C@H], [C@H] [C@H], [C@H]	C, C, (, C, ), C, (, C, ), (, O, ), (, C, C, (, C, ), C, ), C, (, C

## Data Availability

The data used to support the findings of this study are included within the article.

## Supplementary Materials

The following supporting information can be downloaded at https://www.mdpi.com/article/10.3390/ijms26041681/s1.
